# Inhibition of Nuclear Factor-κB Activation in Pancreatic β-Cells Has a Protective Effect on Allogeneic Pancreatic Islet Graft Survival

**DOI:** 10.1371/journal.pone.0056924

**Published:** 2013-02-21

**Authors:** Roy Eldor, Roy Abel, Dror Sever, Gad Sadoun, Amnon Peled, Ronit Sionov, Danielle Melloul

**Affiliations:** 1 Department of Endocrinology, Hadassah University Hospital, Jerusalem, Israel; 2 Goldyne Savad Institute of Gene Therapy, Hadassah University Hospital, Jerusalem, Israel; 3 Department of Biochemistry and Molecular Biology, IMRIC, Hebrew University-Hadassah Medical School, Jerusalem, Israel; University of Bremen, Germany

## Abstract

Pancreatic islet transplantation, a treatment for type 1 diabetes, has met significant challenges, as a substantial fraction of the islet mass fails to engraft, partly due to death by apoptosis in the peri- and post-transplantation periods. Previous evidence has suggested that NF-κB activation is involved in cytokine-mediated β-cell apoptosis and regulates the expression of pro-inflammatory and chemokine genes. We therefore sought to explore the effects of β-cell-specific inhibition of NF-κB activation as a means of cytoprotection in an allogeneic model of islet transplantation. To this end, we used islets isolated from the ToI-β transgenic mouse, where NF-κB signalling can specifically and conditionally be inhibited in β-cells by expressing an inducible and non-degradable form of IκBα regulated by the tet-on system. Our results show that β-cell-specific blockade of NF-κB led to a prolonged islet graft survival, with a relative higher preservation of the engrafted endocrine tissue and reduced inflammation. Importantly, a longer delay in allograft rejection was achieved when mice were systemically treated with the proteasome inhibitor, Bortezomib. Our findings emphasize the contribution of NF-κB activation in the allograft rejection process, and suggest an involvement of the CXCL10/IP-10 chemokine. Furthermore, we suggest a potential, readily available therapeutic agent that may temper this process.

## Introduction

Although the past decade has witnessed substantial developments in the field of islet transplantation [1], only a fraction of grafted islets survives. The reduction in β-cell mass in the immediate post-transplantation period appears to be due to hypoxia, nutrient deprivation and inflammation at the site of implantation [2], [3]. A variety of approaches have been explored to prevent the apoptotic destruction of islets in the experimental setting and, while promising data has been generated *in vitro*, demonstration of an *in vivo* benefit to islet graft survival has been more elusive (reviewed in [2], [4]). Such attempts have included genetic manipulation of the donor islets with anti-inflammatory and antiapoptotic genes such as Bcl-2 [5], [6], X-linked inhibitor of apoptosis protein (XIAP) [6]-[9] and the suppressor of cytokine signalling 1 (SOCS1) [10], as well as the overexpression of the antiapoptotic A20, which preserved functional islet β-cell mass [11]. Pre-treatment of islets with the caspase inhibitors zDEVD-FMK [12], EP1013 [13] or V5 [14] also improved islet survival.

Since inflammation is a primary contributor to graft loss, inhibiting the pro-inflammatory activity of cytokines could eventually prevent the loss of functional islet grafts. Since the NF-κB/Rel family of transcription factors regulates biological processes ranging from apoptosis to inflammation and innate immunity, attempts have been made to block NF-κB activation in models of islet cell transplantation. In models of syngeneic islet transplantation, pretreatment of donor islets with the NF-κB inhibitor dehydroxymethylepoxyquinomicin (DHMEQ) [15], conditional β-cell inhibition of NF-κB improved hepatic intra-portal engraftments [16], and transplantation of TLR4−/− deficient islets [17] each improved graft survival with reduced islet NF-κB activation. Prolonged graft survival was also observed in an allogeneic model of islet transplantation, but when c-Rel null mice (a lymphoid-predominant member of the NF-κB/Rel family) were used as recipients [18].

To further study the role of NF-κB *in vivo*, we have generated a transgenic mouse model, the ToIβ [19], [20], in which NF-κB activation is specifically and conditionally (±doxycycline/Dox) inhibited in β-cells. We previously showed that these mice were more resistant to MLDS-induced diabetes when the NF-κB pathway was inhibited [19]. Using this model, we present in this study further evidence that NF-κB blockade prolonged islet survival in an allotransplantation model, with increased preservation of the engrafted islets under the kidney capsule, and suggest the involvement of the chemokine CXCL10/IP-10 in this process.

## Research Design and Methods

### ToI-β Transgenic Mice

The generated ToI-β transgenic mouse model was previously described [19]. It carries the nondegradable IκBα and luciferase genes (ΔNIκBα-luciferase), regulated by a tetracycline-responsive element, and the reverse tetracycline transactivator (rtTA) under the control of the rat insulin II promoter (RIP7-rtTA). The mouse was generated by cross-breeding two transgenic mouse lines, one carrying the ΔNIκBα-Luciferase genes in an in-house agouti strain of C57B/6:H2^b^ X BALB/c:H2^d^ backgrounds, and the other RIP7-rtTA in a predominantly C3H:H2^k^ background. The transgenic mouse was then inbred for over 20 generations to produce the ToI-β inbred strain. The transgenes were activated *in vivo* by administering 2 mg/ml of doxycycline (Dox; Taro, Israel) in the drinking water. All animals were maintained in a specific pathogen-free research animal facility, and the experiments were approved by the Hebrew University Institutional Animal Care and Use Committee and conducted in accordance with local ethical guidelines.

### Isolation and Culture of Mouse Pancreatic Islets

ToI-β mouse islets were isolated and cultured as previously described [19], in the presence or absence of mouse recombinant cytokines IL-1β (50 U/ml) and INF-γ (1,000 U/ml) that were purchased from R&D Systems Inc. (Minneapolis, MN, USA).

### Medium Nitrite-concentration Measurement

ToI-β isolated islets were preincubated for 24 h with Bortezomib (BZB) (100 nM, LC laboratories, Woburn, MA, USA) [21] before the cytokines were added for an additional 48 h. Medium (100 µl) from the islet cultures containing 200 islets**/**ml were added to an equal volume of Greiss reagent, as previously described [19].

### Islet Transplantation

Prior to transplantation, 10-week-old recipient mice were rendered diabetic by a single intra-peritoneal injection of streptozocin (250 mg/kg) (Sigma, St Louis, MO, USA), reaching consecutive glycemic values of >360 mg/dl: Control 441.9+/−37.6 mg/dl; Dox 417+/−29.2 mg/dl; BZB 427.25+/−30.1 mg/dl.

Mice were anesthetized with isoflurane (Nicholas Piramal India Ltd, Mumbai, India) and 500 islets were transplanted under the kidney capsule [8], [14], [22]. In the allogeneic experiments, SJL (H2^s^) mice were used as recipients since they do not share any of the MHC H2 haplotypes of the original double-transgenic Tol-β. In syngeneic islet transplantations, donor and recipient Tol-β inbred mice were from the same litter. The postoperative follow-up was conducted by daily measurements of blood glucose levels using an Accu-Check Performa glucose meter (Roche, Mannheim, Germany). Islet grafts were considered functional when the measurements of non-fasting blood glucose were below 200 mg/dl for at least 5 consecutive days after transplantation, whereas graft rejection was defined as a return to hyperglycemia when consecutive glycemic levels were higher than 200 mg/dl. In another set of transplantations, 1 mg/kg of the proteasome inhibitor Bortezomib was injected intraperitoneally twice weekly [21] until graft rejection.

For histological analysis of relative endocrine/graft area, the kidneys containing grafted islets of untreated and Dox-treated recipients were retrieved on day 7 post-transplantation, then paraffin-embedded and sectioned longitudinally at a thickness of 5 µm to encompass as much of the graft as possible. Sections were conventionally stained with hematoxylin-eosin and photographed at a 10X magnification with identical exposure times, using an Olympus BX41 microscope and the Olympus DP71 camera. The processed images were combined to create a whole picture of the graft while omitting the kidney tissue and then analyzed using the software ImageJ (Freeware, NIH, Bethesda, MD, USA). A standardized point-counting grid was superimposed on the reconstituted engrafted picture and the number of intersections containing endocrine tissue was compared to the total graft area (i.e., the total number of grid points hitting the structure of interest being counted). The grid technique has been often used to measure beta cell mass in a similar fashion [23], [24]. Immunohistochemistry of insulin was performed using Histostain Plus Broad Spectrum (Invitrogen, Frederick, MD), according to manufacturer instructions, on the graft-bearing kidneys sections. Guinea pig anti-insulin serum (Linco, Seaford, DE) or anti-mouse IP-10 antibody (R&D systems, Minneapolis, MN, USA) were used as primary antibodies followed by incubation with the secondary antibodies HRP-conjugated rabbit anti-guinea pig or HRP-streptavidin, respectively ((Zymed, San Francisco, CA, USA).

For gene expression analysis, the syngeneic grafts were retrieved 24 h post-transplantation and mRNA levels were assessed by real-time PCR using the primers listed in Supplementary Material. The results presented solely include those obtained from the grafts containing less than 5% of kidney tissue contamination as determined by the levels of the transcripts of novel kidney gene (NKT) in the retrieved islets.

### Data Presentation and Statistical Analysis

The data are presented as the mean ± SEM. Statistical analysis of medium nitrite concentration, average graft survival and relative endocrine/total graft area was performed using the paired Student’s *t*-test. Post-transplantation graft survival was assessed by Kaplan-Meier analysis and comparison of survival curves by logrank test using MedCalc 12.3.0.0. In all tests, *p*<0.05 was considered statistically significant.

## Results

### Temporal Inhibition of NF-κB Activation in Pancreatic β-cells Improves Allogeneic Islet Graft Survival

**Figure 1:**
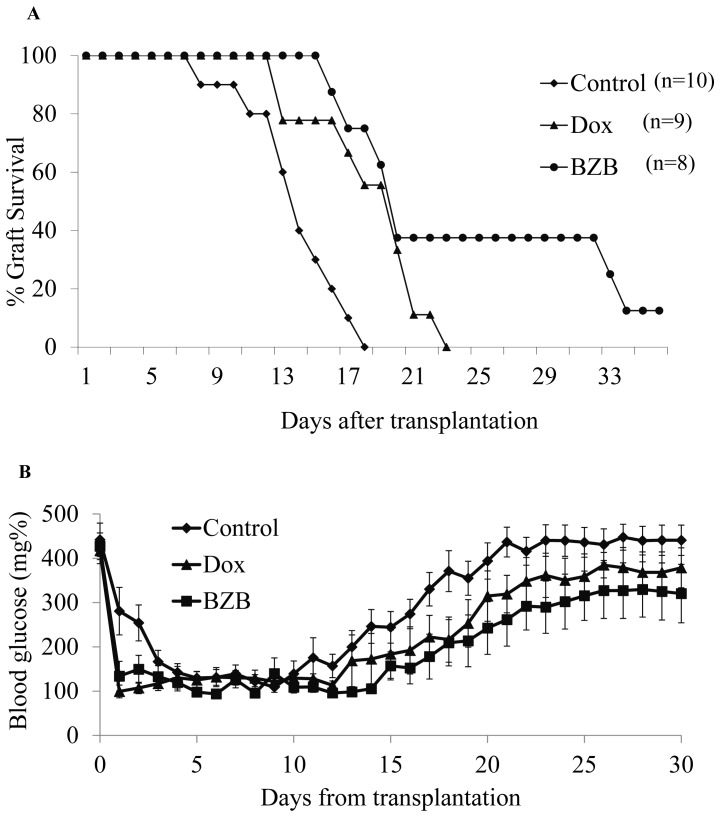
Survival of ToI-β pancreatic islet grafts in allogenic SJL- mice. 500 ToI-β islets were transplanted under the kidney capsule of allogeneic SJL mice. Rejection/graft survival was determined by tail blood glucose measurements. Graft rejection was defined as consecutive measurements of glycemia >200 mg/dl. (**A**) Untreated control mice (diamonds, n = 10); mice that were exposed to Dox 24 hours after transplantation (triangles, n = 9); and mice that were exposed to Bortezomib 24 hours after transplantation (circles, n = 8). Statistical analysis was done by Kaplan-Meier estimation and comparison of survival curves by the MedCalc logrank test. p = 0.004 Dox *vs* control, p = 0.001 BZB *vs* control. (**B**) Blood glucose of ToI-β islet grafts in untreated SJL control mice (black diamonds); mice exposed to Dox (triangles) and mice treated with Bortezomib (BZB, squares). Blood glucose areas under the curve (AUC) were smaller in treated *vs* untreated recipient mice: Dox *vs*. Control *p* = 0.025, BZB *vs* Control *p* = 0.01.

To determine the role of NF-κB activation in islet graft rejection, 500 isolated islets from ToI-β mouse donors were transplanted into the diabetic allogeneic recipient SJL mouse strain. The SJL (H2^s^) was chosen as it does not share any of the MHC H2 haplotypes of the original double-transgenic Tol-β mouse line (H2^b^; H2^d^; H2^k^). Transplantation of untreated ToI-β islets (control) resulted in a swift recovery of normoglycemia, which was maintained for an average of 13.9 days before graft failure (FIG. 1A, Control; n = 10). Similar results were obtained when donors, recipients and islet grafts were exposed to Dox in the drinking water and in the culture media a few days prior to transplantation (data not shown). However, a modest but significant delay in graft failure of 4.5 days was achieved when Dox was introduced to the drinking water of recipient mice 24 hours after the transplantation (FIG. 1A, Dox; average days to rejection: 18.4; n = 9; *p = *0.004 Dox *vs* Control).

### Systemic Proteasome Inhibition Prolongs Allogeneic Islet Graft Survival

**Figure 2:**
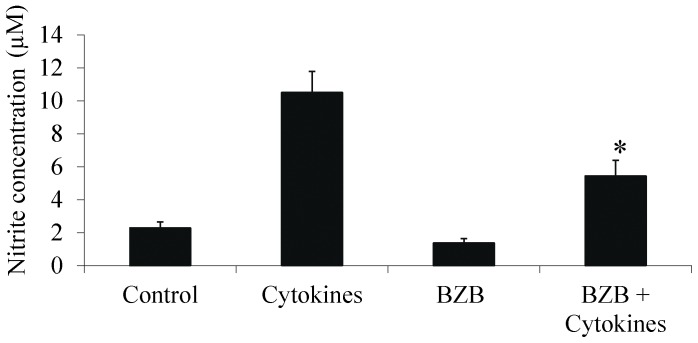
Medium nitrite levels secreted from islets exposed in vitro to IL-1β (50 units/ml) and IFN-γ (1,000 units/ml) for 48 h in the presence or absence of Bortezomib (BZB- 100 **nM).** Nitrite data are pooled from two separate experiments incorporating 5–6 repeats of each treatment, presented as the mean ± SEM. *p value = 0.023 for BZB+Cytokines *vs* Cytokines.

One of the major problems with employing genetically modified strategies or using viral vectors for transfection is the difficulty in expressing genes in primary islets. For possible future clinical use, we propose as a proof of concept the use of the known proteasome inhibitor Bortezomib (BZB) [21]. We initially tested its effect in vitro, exposing ToI-β islets to a combination of IL-1β+IFN-γ cytokines for 48 h and measuring nitrite secretion into the media. FIG. 2 demonstrates a reduction in cytokine-induced NO secretion with Bortezomib. Interestingly, a reduction of about 50% in cytokine-stimulated NO secretion was observed when NF-κB activation was inhibited, whether by using Bortezomib or by activation of the endogenous super-repressor IκB transgene in Dox-treated ToI-β islets (as previously demonstrated in Eldor et al [19]).

Similar allogeneic transplantation experiments were conducted in which the ToI-β islets were transplanted into diabetic allogeneic recipient SJL mice, which were injected with Bortezomib 24 h post-transplantation. FIG. 1A clearly shows that BZB led to a delay in graft rejection greater than that displayed in the Dox-treated model (BZB; average day of rejection: 26.5; n = 8; *p* = 0.001 BZB *vs* Control). The benefit of Dox- or Bortezomib-treatment was demonstrated by the decrease in blood glucose levels compared with untreated controls (FIG. 1B). When the glucose area under the curve (AUC) was calculated, treated mice had a significantly lower AUC compared with control animals (Dox *vs*. Control *p* = 0.025, BZB *vs* Control *p* = 0.01).

### Post-transplantation NF-κB Inhibition Leads to Preservation of Endocrine Mass in the Islet Graft

**Figure 3:**
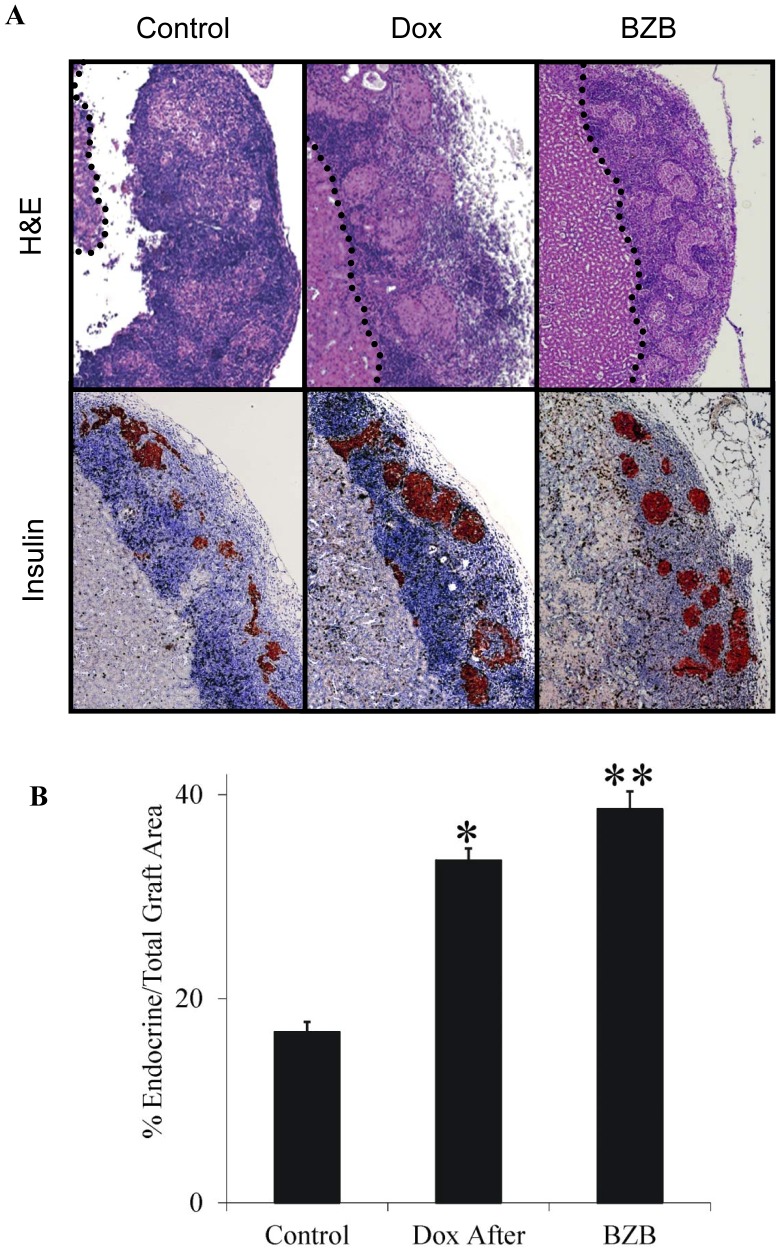
Inhibition of NF-κB activation is associated with an increased endocrine/total graft area ratio in the islet graft. **A.** The recipient graft-bearing kidneys from normoglycemic untreated mice, and Dox- or Bortezomib (BZB)-treated animals were removed 7 days after allogeneic transplantation, fixed in formaldehyde, thin-sliced and stained with hematoxylin/eosin solution. The border between the kidney and the graft is marked (Upper panel). Paraffin sections were stained for insulin (Lower panel). **B.** Using a fixed grid, the percentage of endocrine area was calculated from the total graft area. Results are the average of at least five non-consecutive sections incorporating the whole graft area. *p = 0.033 Dox *vs* control; **p = 0.0001 BZB *vs* control n = 3.

To further study the role of NF-κB in the islet transplantation model, we sought to determine the relative endocrine *vs* total graft area. Measurements were taken 7 d post-transplantation, a point at which grafts were still fully functional, as evidenced by normoglycemia measured at this point and yet, sufficient time had elapsed for the development of an immune rejection response [25], FIG. 3 depicts a grid estimate of the relative area of endocrine tissue as a percentage of the total graft area in grafts retrieved from untreated mice (Control), and from mice exposed to Dox or BZB beginning 1 day post-operation. Since the mice were transplanted with the exact same number of 500 islets, this may be considered a rough estimate of the remaining β-cell/endocrine mass in the graft. There was greater preservation of the relative endocrine area in the Dox- and BZB-treated mice *vs* control mice (*p* = 0.033 Dox *vs* control; *p* = 0.0001 BZB *vs* control; n = 3), which correlated with a better preservation of islet graft structure and of insulin positive cells.

### Potential Role of IP-10/CXCL10 in NF-κB-mediated Early Protection and Delay in Islet Graft Rejection

**Figure 4:**
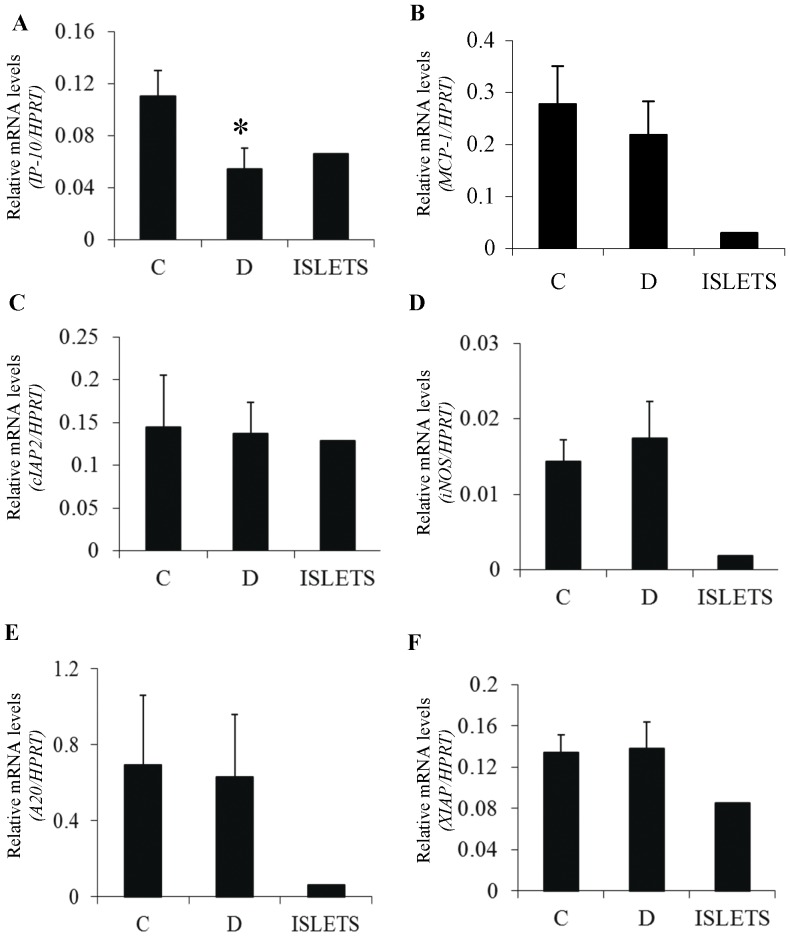
Real-time PCR analysis of NF-κB target genes in islet grafts 24 hours after transplantation. CXCL-10/IP-10 (*p = 0.03 Dox *vs* control) (**A**), MCP-1 (**B**), cIAP2 (**C**)**,** iNOS (**D**), A20 (**E**) and XIAP (**F**). mRNA was extracted from ToI-β islet grafts retrieved from the kidney capsule 24 hours after syngeneic transplantation. Prior to transplantation, islet grafts were exposed to Dox in the culture media for 48 hours (D) or untreated controls (C). The right columns represent relative gene expression in isolated, untreated islets (ISLETS). Results are shown as fold induction normalized to HPRT values. Only retrieved grafts with less than 5% kidney contamination were included in the study, as assessed by expression of the kidney tissue-specific NKT (novel kidney transcript) gene. Results are the mean ± SEM of three to five independent experiments.

In an attempt to further elucidate the effect of NF-κB in the peri-transplantation period, we set out to analyze the expression of putative NF-κB target genes in Dox-treated and untreated grafts. To this end, syngeneic transplantation experiments were performed and the islet transplants were retrieved 24 h after transplantation, at a time of minimal inflammatory cell infiltration. Surprisingly, only IP-10/CXCL10 expression was significantly reduced by the β-cell specific inhibition of NF-κB (FIG. 4A; p = 0.03 Dox *vs* control; n = 5). However, no NF-κB-dependent changes were noted in the expression of genes previously shown to be stimulated by NF-κB activation *in vitro*, including iNOS, MCP-1 and NF-κB-mediated antiapoptotic genes such as A20 and XIAP (FIG. 4B–4F). In all grafts retrieved, expression of the kidney tissue-specific NKT (novel kidney transcript) gene was measured to assess the degree of kidney contamination. Only grafts with less than 5% kidney contamination are presented.

**Figure 5:**
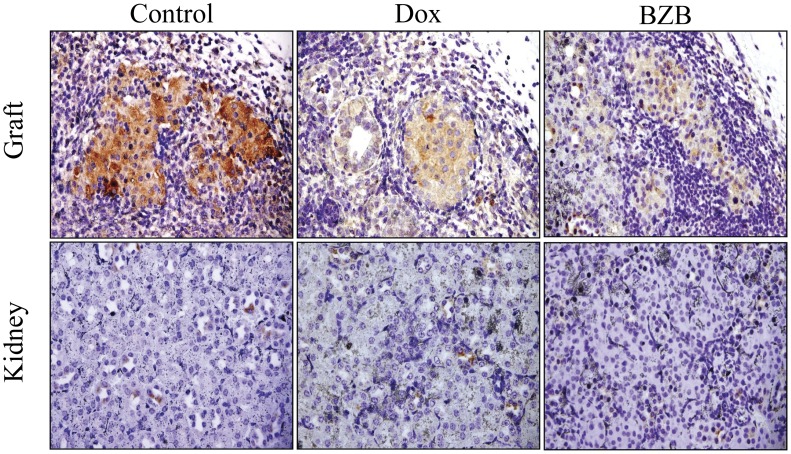
Decreased IP-10 in doxycycline- or Bortezomib-treated islet grafts. Representative anti-CXCL10/IP-10 stained graft-bearing kidney sections from normoglycemic untreated mice (control), Dox- or Bortezomib (BZB)-treated animals, 7 days after allogeneic transplantation (Upper panel- Graft). As noted in control the cytoplasmic IP-10 staining is more intense in control islets than in Dox- or Bortezomib-treated mice. The lower panel represents IP-10 staining of kidney sections of the corresponding graft adjacent areas.

To confirm the effect of NF-κB blockade, or that of BZB on CXCL10/IP-10 expression, immunohistochemical staining of graft sections of kidneys collected 7 d post-transplantation from normoglycemic recipient mice, was performed. As shown in FIG. 5, CXCL10/IP-10 cytoplasmic staining was markedly decreased in both islet grafts originated from Dox- or BZB- treated recipients when compared with untreated animals.

Thus NF-κB mediated IP-10 expression may be involved both during engraftment and, at later stages in attracting inflammatory cells. These results highlight that the NF-κB-mediated activation of IP-10 could be a potential immediate target chemokine for islet graft survival and function in the peri-transplantation period.

## Discussion

The aim of our study was to investigate and test a strategy to induce cytoprotection following allogeneic islet transplantation. The current results demonstrate a delay in islet graft rejection when the NF-κB pathway was specifically inhibited in β-cells after transplantation. These results concur with preservation of the endocrine graft area (*vs* immune infiltrate) in Dox-treated *vs* untreated control mice as observed 7 d after transplantation, suggesting that NF-κB activation in β-cells could play a role in immune-mediated early islet graft rejection.

To explore a possible therapeutic option, the proteasome inhibitor Bortezomib was administered to recipient animals following islet transplantation. The systemic treatment resulted in further prolonged islet allograft survival, with histological preservation of the endocrine area. These results are in accordance with a recent report demonstrating a delay in porcine islet graft rejection transplanted into athymic nu/nu mice after a single injection of the IKK-β inhibitor, BMS-345541 [26]. In fact, pharmacological inhibitors of the proteasome not only inhibit the activation of NF-κB in islet cells, but they also affect the function and survival of immune cells leading to reduced inflammatory manifestations in several models of immune-mediated disorders [27]. Bortezomib is currently approved for therapeutic use in multiple myeloma, with relatively low toxicity, and can therefore potentially be used as an immune modulator in the islet post-transplant period.

The transcription factor NF-κB has been shown to regulate the expression of numerous genes that play important roles in cellular stress responses, cell growth, survival and apoptosis [28]-[30]. Numerous reports, including our own, have shown that sustained activation of NF-κB is an important cellular signal in initiating the cascade of events culminating in β-cell death [31]-[38]. Transfection of an NF-κB “superrepressor” gene into MIN-6 insulinoma cell lines, human CM and NES2Y β cell lines, INS-1E cells, purified rat islet β cells, and human and mouse islets led to their protection from cytokine-induced, NF-κB-mediated apoptosis *in vitro* [32], [39], [40]. Similar results were obtained when NF-κB inhibition was achieved through transduction of human and mouse islets by a cationic peptide transduction domain. This in turn mediates delivery of a peptide inhibitor of IκB kinase [derived from IKKβ (NBD; Nemo-binding domain)], and completely blocks the detrimental effects of IL-1β on islet function and viability [41].

Nevertheless, a few several reports have challenged these observations by demonstrating a possible anti-apoptotic effect of NF-κB activation in certain experimental settings. In a model of overexpression of cFLIP [the cellular FLICE (FADD-like IL-1 beta-converting enzyme)-inhibitory protein] in β-TC-Tet cells, cytokine-dependent apoptosis was prevented concomitantly with increasing basal and IL-1β-mediated transcriptional activity of NF-κB [42]. In another model, siRNA-mediated gene silencing of NF-κB in INS-1E cells led to reduced iNOS and NF-κB gene expression but did not prevent cytokine-induced apoptosis [43]. Furthermore, exposure of β-cells to the tyrosine kinase inhibitor imatinib mesylate was associated with NF-κB activation and reduced β-cell apoptosis. The anti-apoptotic effect disappeared in the presence of an NF-κB inhibitor [44]. Additional observations indicated that stimulation of NF-κB protects β-cells from TNF-α-mediated apoptosis [45]. At low concentrations of IL-1β, and following exposure for a limited period of time, NF-κB activation by the cytokine increases β-cell replication and decreases apoptosis [46]. Finally, it has been suggested that transient and moderate laminin-5-rich extracellular-matrix activation of NF-κB may be essential to maintaining proper β-cell glucose-stimulated insulin secretion [47]. A possible reason for inconsistency may be the result of a more rapid, marked, and non-oscillatory activation of NF-κB in β-cells exposed to cytokines as opposed to other cell types (e.g., fibroblasts) [39].

It is thus reasonable to postulate that, under physiological circumstances, the cytokine produced locally by islet cells, including β-cells, by activating NF-κB may play a role in maintenance of β-cell mass and survival [48]. However, under pathophysiological conditions, activation of NF-κB induces the expression of genes whose products can lead to β-cell apoptosis.

We further analyzed the expression of a selected set of putative NF-κB target genes in Dox-treated and untreated mice 24 h after transplantation in a syngeneic islet transplantation model. Surprisingly, only IP-10/CXCL10 expression was significantly reduced by β-cell-specific NF-κB inhibition, though this is consistent with the robust NF-κB-dependent increase in IP-10 secretion we previously found in ToI-β islets exposed to inflammatory cytokines [19]. A growing body of evidence has shown that there is an association between the levels of the chemokine IP-10/CXCL10 in various tissues and the inflammatory/immune processes occurring during organ transplantation [49]. Importantly, IP-10 was recently detected in the insulin-producing cells in islets of recent-onset T1D patients, and its receptor CXCR3 was associated with the infiltrating T cells in the islet area [50]. Similarly, neutralizing IP-10 antibodies suppressed the occurrence of cyclophosphamide-induced diabetes in NOD mice [51]. In this model, IP-10 expression was first being detected in β-cells in the peri-insulitis stage, and the levels gradually increased as the degree of insulitis progressed to the stage of intra-islet insulitis. Similar observations were made in the present study using ToI-β islet allografts, where a correlation was noticed between the IP-10 staining intensity in the islet grafts and the blockade of NF-κB pathway i.e. treated *vs* untreated mice (data not shown). Finally, in an allogeneic model of pancreatic islet transplantation, CXCR3−/− recipients or post-transplantation administration of anti-IP-10 antibodies inhibited T-cell trafficking to the graft site and prolonged islet survival [25]. Interestingly, this extended period of islet survival was similar to that observed in our experimental model, in which the NF-κB pathway was specifically inhibited in β-cells. These results suggest that IP-10 may be a potential contributor to the NF-κB-mediated inflammatory/immune mechanism.

In conclusion, we present evidence for a beneficial therapeutic effect of NF-κB inhibition in islet transplantation, and suggest NF-κB-mediated IP-10 expression as an early activated chemokine, involved in islet graft survival and function in the post-transplantation period.
